# Resilience in patients with post-traumatic stress disorder

**DOI:** 10.1192/j.eurpsy.2025.1943

**Published:** 2025-08-26

**Authors:** R. Barbarić, S. Burić

**Affiliations:** 1 Psychiatry, University clinical hospital, Mostar, Bosnia and Herzegovina

## Abstract

**Introduction:**

Resilience was first introduced into psychology and psychiatry from technical sciences and afterwards thorough medicine and healthcare. It represents a complex set of various protective and salutogenic factors and process important for understanding health and illness, and treatment and healing processes. It is defined as a protective factor that makes an individual more resilient to adverse events that lead to positive developmental outcomes. Resilience is a positive adaptation after stressful situations and it represents mechanisms of coping and rising above difficult experiences, i.e., the capacity of a person to successfully adapt to change, resist the negative impact of stressors and avoid occurrence of significant dysfunctions. It represents the ability to return to the previous, so-called “normal” or healthy condition after trauma, accident, tragedy, or illness. In other words, resilience refers to the ability to cope with difficult, stressful and traumatic situations while maintaining or restoring normal functioning. The higher the resilience, the lower the vulnerability and risk of illness (Babic R, et al. Psychiatria Danubina 2020; Vol. 32, Suppl. 2, 226-232). In the development of post-traumatic stress disorder (PTSD), there is a positive association with negative emotions, on the other hand, PTSD symptoms are negatively correlated with positive emotions (Deborah JC. TD collection for University of Nebraska – Lincoln, 2001), which often depends on resilience which is inversely proportional to the onset of PTSD and as such plays an important role in treatment of anxiety disorders, depression and stress reaction (Green et al. Assessment 2014; 21:443-5, Connell et al. Fr J Psychiatry (Johannesburg) 2013; 19:16, Zerach et al. sr J Psychiatry Relat Sci 2013; 50:91-98).

**Objectives:**

The aim of this research was to investigate the connection between resilience and post-traumatic stress disorder.

**Methods:**

Socio-demographic questionnaire (personal creation).

Resilience questionnaire – Croatian version (Connor-Davidson Resilience Scale 25).

Clinical questionnaire for ptsd, diagnostic version for current and lifetime PTSD - Croatian version (Clinician Administered PTSD Scale, CAPS-DX).

**Table:** 

CAPS-DX (PTSD)	Resilience	
	r	p
Reexpiriencing symptoms	-0.446	<0.001
Avoidance symptoms	-0.561	<0.001
Arousal and reactivity symptoms	-0.509	<0.001
CAPS-DX total	-0.542	<0.001

**Results:**

**Image:**

**Figure:**
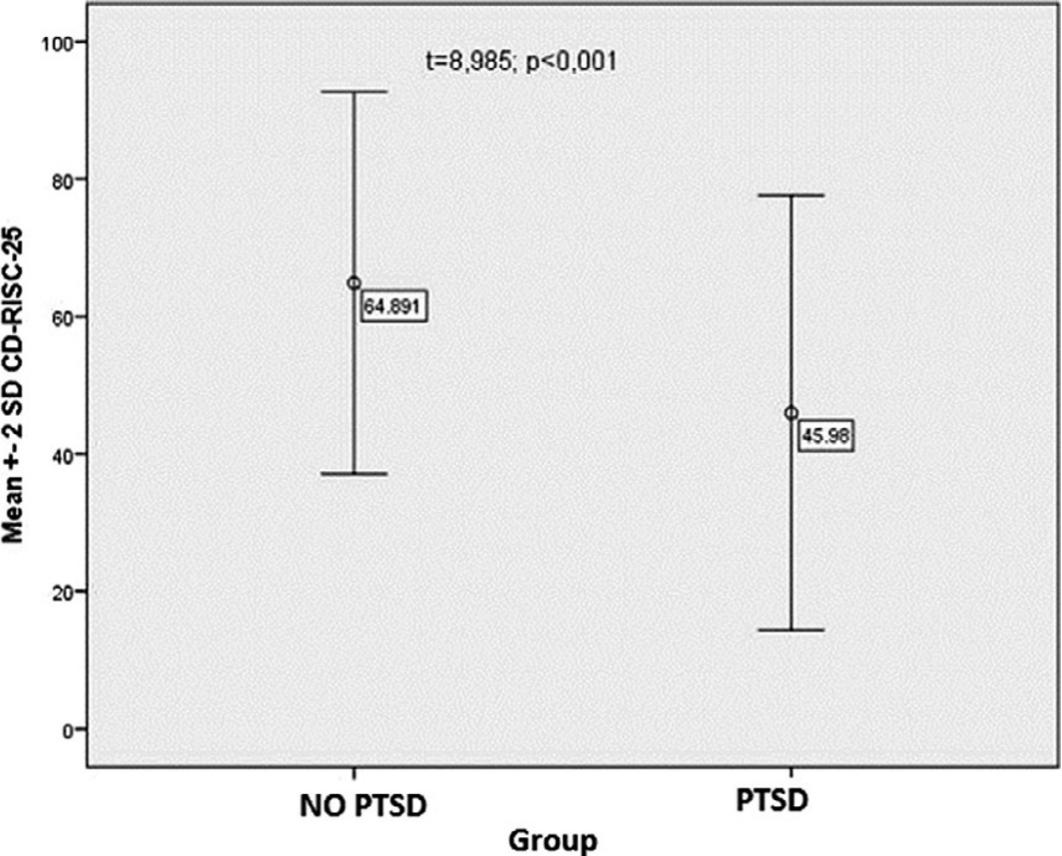

**Image 2:**

**Figure:**
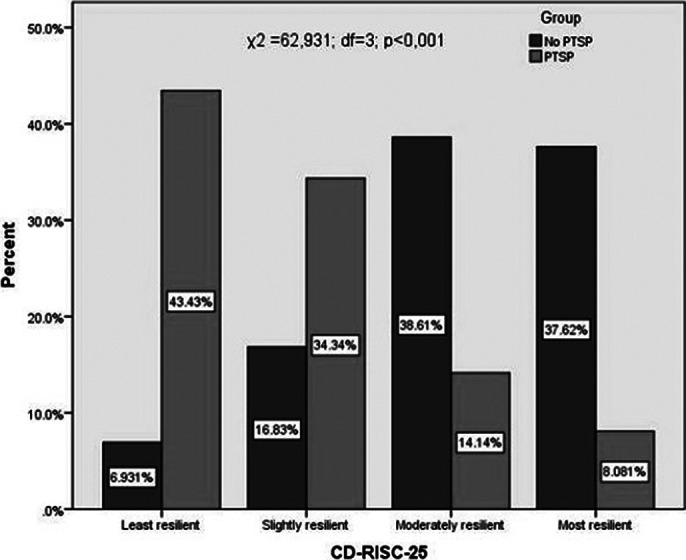

**Conclusions:**

The level of resilience had an impact on the onset, development and outcome of PTSD.

People without PTSD show a statistically higher level of resilience compared to respondents with PTSD.

Respondents without PTSD are statistically significantly more represented in the groups with moderately high resilience, while those with PTSD are the most in the group with the least resilience.

**Disclosure of Interest:**

None Declared

